# Chronic unilateral chemosis following the use of amlodipine besylate

**DOI:** 10.1186/1471-2415-14-124

**Published:** 2014-10-21

**Authors:** Kyeong Hwan Kim, Wan Soo Kim

**Affiliations:** Department of Ophthalmology, Inje University Haeundae Paik Hospital, Busan, South Korea; Department of Ophthalmology, Inje University College of Medicine, Busan, South Korea; Department of Ophthalmology, Inje University Busan Paik Hospital, Busan, South Korea

**Keywords:** Amlodipine, Calcium channel blocker, Chemosis, Conjunctiva, Edema

## Abstract

**Background:**

This case report describes a patient with chronic unilateral chemosis, likely due to treatment with amlodipine besylate.

**Case presentation:**

A 52-year-old man visited the clinic with symptoms of foreign body sensation and puffiness in his right eye, which had persisted for 4 months. There were no other symptoms, such as itching and pain, in his right eye and no specific symptoms in his left eye. He had been treated for hypertension and hyperlipidemia for the previous 5 months with once daily amlodipine besylate/atorvastatin (Caduet) and candesartan cilexetil (Atacand). Examination revealed marked swelling of the inferior bulbar conjunctiva in the right eye. Evaluation revealed no specific causes for the longstanding chemosis. A change of medication to telmisartan/hydrochlorothiazide (Micardis Plus) without amlodipine besylate resulted in significant improvements in his symptoms after 1 month and complete remission after 8 months.

**Conclusion:**

Prior to invasive evaluation including biopsy, specific drugs should be considered as possible causes of idiopathic longstanding conjunctival chemosis.

## Background

Conjunctival chemosis—the presence of excess fluid in the conjunctival interstitium—is caused by several conditions, including exposure, trauma, infection, allergy, obstruction of lymphatic and venous outflow, and inflammation of the conjunctiva and adjacent structures. This symptom is usually self-limiting or shows complete recovery following removal of the causal factor. However, in some patients swelling of the conjunctiva may persist for over 6 months with no obvious cause. In some idiopathic cases, tissue inflammation or lymphangiectasia may give rise to irreversible chronic chemosis [1].

Amlodipine besylate is a calcium channel blocker (CCB), one of the most commonly used classes of antihypertensive agents. Peripheral edema is a common dose-dependent adverse effect of amlodipine besylate and a major cause of discontinuation of therapy [2]. We describe here a patient with chronic unilateral chemosis, likely due to treatment with amlodipine besylate.

## Case presentation

A 52-year-old man visited our clinic with symptoms of foreign body sensation and puffiness in his right eye, which had persisted for 4 months. He was originally diagnosed with allergic conjunctivitis by a general ophthalmologist and was treated with olopatadine eye drops (Pataday; Alcon, USA) once daily and 1% prednisolone eye drops (Pred-Forte; Allergan, USA) four times per day for 3 weeks. He was referred to our clinic after the treatment was unsuccessful, even after oral prednisolone 30 mg/day for 3 days with a tapering schedule was added to topical therapy for an additional one week.

Upon referral to our clinic, the patient stated that his symptoms were negligible when he woke up in the morning, but gradually increased over several hours and persisted with no fluctuations during the day time. There was no itching, pain, or other symptoms in his right eye and no specific symptoms in his left eye. His medical history included hypertension and hyperlipidemia, for which he had been treated for the previous five months with once daily amlodipine besylate 5 mg/atorvastatin 10 mg (Caduet; Pfizer Inc, USA) and once daily candesartan cilexetil 8 mg (Atacand; AstraZeneca, UK), respectively. He reported no history of other systemic diseases, drug allergy, atopic disease or trauma, but he reported always sleeping on his right side. Physical examination revealed no other specific findings such as pitting edema.

**Figure 1:**
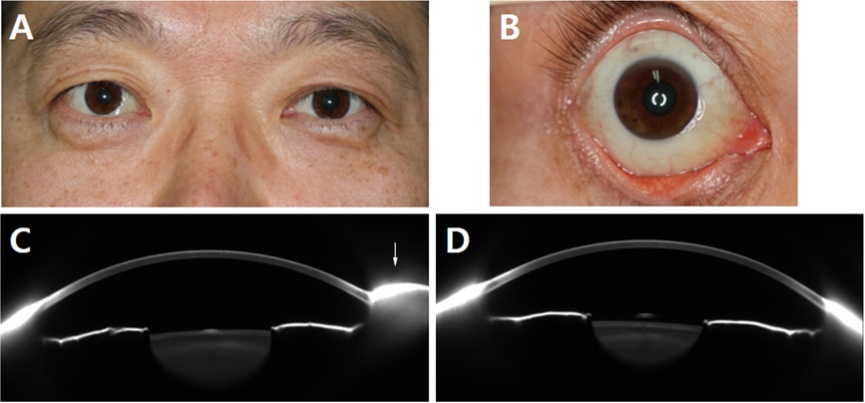
**Clinical photograph and Scheimpflug images at presentation.** Clinical photographs showing conjunctival chemosis in the right eye **(A)**. Swelling started in the interpalpebral area, gradually increasing toward the inferior half and leaving a normal superior bulbar conjunctiva **(B)**. Scheimpflug images at a 90-degree vertical scan showing marked conjunctival swelling inferiorly (arrow) in the right eye **(C)**, although the conjunctiva of the left eye remained unchanged **(D)**.

Slit-lamp examination showed moderate conjunctival chemosis in his right eye. Swelling was gravity dependent, starting in the interpalpebral area and gradually increasing toward the inferior half, leaving a normal superior bulbar conjunctiva (Figure 1A and B). The bulbar conjunctiva showed no evidence of a dilated lymphatic channel, and the palpebral conjunctiva showed no papillary reaction or follicular hypertrophy. Both eyes showed no evidence of conjunctival hyperemia or injection, and his left eye was normal. Vision was 20/20 in each eye and intraocular pressure, measured by Goldmann applanation tonometry, was 16 mmHg in his right eye and 18 mmHg in his left eye. Exophthalmometric measure was normal, and neuroimaging using magnetic resonance imaging with contrast enhancement showed no abnormalities such as an orbital mass or venous or lymphatic obstruction. Scheimpflug imaging revealed marked swelling of the inferior bulbar conjunctiva in the right eye, but the anterior chamber depths of the two eyes did not differ significantly (Figure 1C and D).

**Figure 2:**
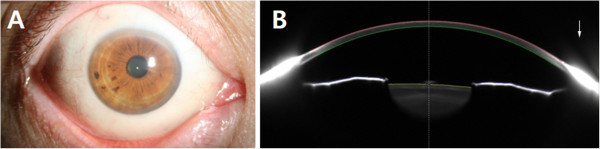
**External appearance and Scheimpflug image after the cessation of the amlodipine besylate.** Slit-lamp examinations after the change of medication showed that conjunctival swelling in the right eye had completely remitted (arrow) one month later **(A)** and remained stable until the last follow up visit 8 months later. Scheimpflug image at a 90-degree vertical scan also revealed normal appearing conjunctiva **(B)**.

Serum IgE and antibody titers for grass and house dust mites were normal. Blood tests, including renal and thyroid function tests, protein concentration, inflammatory markers such as erythrocyte sedimentation rate and C reactive protein, complete blood count and serum chemistry, showed results within normal ranges. The patient had not received any other topical or systemic treatment since the referral from the general ophthalmologist. After the patient was referred to the department of medicine for evaluation of allergic and other systemic diseases, his antihypertensive medication was changed to telmisartan 40 mg/hydrochlorothiazide 12.5 mg (Micardis Plus; Boehringer Ingelheim, Germany). Slit-lamp examination 1 month later showed an almost complete absence of conjunctival chemosis, and he reported that his symptoms were much improved. Therefore, the patient was followed up without any ocular treatment. All of his symptoms had completely remitted and remained stable at his last follow-up visit, 8 months after the change in systemic medication (Figure 2).

## Discussion and conclusion

Unilateral conjunctival chemosis persisted in the patient described here for about 4 months, during which time we were able to exclude several possible causes, including exposure, trauma, infection, obstruction of lymphatic or venous drainage, inflammation, or systemic disease, resulting in fluid overload. Previously, tissue inflammation or lymphangiectasia was observed in a biopsy specimen from a patient with idiopathic chronic conjunctival chemosis [1]. In contrast, we could exclude an inflammatory cause in our patient, since serum markers of inflammation were within normal range, long term treatment with local and systemic steroids failed to improve symptoms, and combined medications such as atorvastatin and an angiotensin II receptor blocker (ARB) are reported to have anti-inflammatory properties [3, 4]. If structural abnormalities are present, chemosis should be irreversible. However, we found that chemosis was reversible after amlodipine besylate was discontinued, indicating that chemosis in this patient resulted from the medication rather than from any structural abnormality.

CCBs have been reported to preferentially dilate arterioles, increasing the pressure gradient between arteriolar and venule capillaries and leading to the extravasation of intravascular fluid and resultant vasodilatory edema [2]. Since the condition of the our patient resolved completely after discontinuing amlodipine besylate, and assays for other causes yielded uniformly negative results, the conjunctival chemosis observed in the patient was likely due to CCB-induced vasodilatory edema. Our patient was not rechallenged with amlodipine after resolution of chemosis for ethical reasons and because his symptoms had resolved completely and his blood pressure was controlled on a new medication. A low dose of diuretics, administered after amlodipine discontinuation, likely did not affect the resolution and progression of chemosis, inasmuch as CCBs are reported to have a natriuretic effect and edema is thought to result from increased intracapillary pressure rather than from salt and water retention [5].

To our knowledge, only one other case report is similar to the case described here [6]. However, our patient was treated with 5 mg/day amlodipine as the starting and lowest dose, with no increase in dosage. At this dose, only approximately 5% of patients will experience swelling of peripheral tissue. Nevertheless, our patient developed conjunctival edema despite combination treatment with ARB, which reduces CCB-induced vasodilatory edema [2]. Moreover, in contrast to the previous patient [6], our patient experienced conjunctival chemosis without peripheral edema.

Edema in only one eye without peripheral edema may have several causes. The preferential arteriolar vasodilating activity of CCBs has been shown to increase capillary pressure and flow, increasing capillary permeability and fluid hyperfiltration [7]. Since our patient always slept in the right lateral decubitus position, interstitial fluid on the right side of his body, including his right eye, may have increased while sleeping in this position. In addition, capillary fluid filtration is held constant by the veno-arteriolar reflex during a positional change to upright position, causing postural vasoconstriction in both the arteriolar and the venous limb in normal situations [2]. However, attenuation of this homeostatic mechanism due to selectively diminished precapillary vasoconstriction by CCBs could augment interstitial fluid accumulation on the right side of the body after awakening. Furthermore, CCBs activate both the sympathetic nervous system and the renin-angiotensin system [8]. Thus, although CCBs limit precapillary vasoconstriction by the sympathetic nervous system, postcapillary vasoconstriction by the renin-angiotensin system is buffered by ARBs, normalizing intracapillary pressure and avoiding peripheral edema [2]. The greater sensitivity of conjunctival vessels to angiotensin II than to phenylephrine [9] may have resulted in conjunctival chemosis in the absence of peripheral edema. That is, the ARB may have buffered postcapillary vasoconstriction less effectively and the veno-arteriolar reflex occurring during a positional change may be less prominent in conjunctival than in peripheral capillaries. Although the diffuse accumulation of interstitial fluid in the right eye during sleep may have been asymptomatic initially, a lack of postural vasoconstriction and less effective buffering by ARB may have augmented gravity dependent fluid accumulation in the right eye, inducing symptoms during the daytime. Studies assessing the differences in vascular properties and neurotransmitter receptors between conjunctiva and other tissues are necessary to determine the mechanism underlying the induction by amlodipine besylate of edema in only one eye without peripheral edema, including swelling of the feet or ankles.

In summary, we have described a patient with chronic unilateral chemosis, which was likely due to amlodipine besylate. Prior to invasive evaluation, including biopsy, specific drugs should be considered as possible causes of idiopathic longstanding conjunctival chemosis. Early identification of the causative drug is important, as the risks of damage and scarring of lymphatic structures increase as the duration of chemosis increases.

### Consent

Written informed consent was obtained from the patient for publication of this case report and any accompanying images. A copy of the written consent is available for review by the Editor of this journal.

## Authors’ information

Presented in part at the annual meeting of Korean Ophthalmological Society, Goyang, Korea, April 2013 and the Asia-Pacific Association of Cataract and Refractive Surgeons, Singapore, July 2013.

## Abbreviations

CCB: Calcium channel blocker
ARB: Angiotensin II receptor blocker.
